# Evolution of High Cellulolytic Activity in Symbiotic *Streptomyces* through Selection of Expanded Gene Content and Coordinated Gene Expression

**DOI:** 10.1371/journal.pbio.1002475

**Published:** 2016-06-08

**Authors:** Adam J. Book, Gina R. Lewin, Bradon R. McDonald, Taichi E. Takasuka, Evelyn Wendt-Pienkowski, Drew T. Doering, Steven Suh, Kenneth F. Raffa, Brian G. Fox, Cameron R. Currie

**Affiliations:** 1 DOE Great Lakes Bioenergy Research Center, University of Wisconsin-Madison, Madison, Wisconsin, United States of America; 2 Department of Bacteriology, University of Wisconsin-Madison, Madison, Wisconsin, United States of America; 3 Department of Biochemistry, University of Wisconsin-Madison, Madison, Wisconsin, United States of America; 4 Department of Entomology, University of Wisconsin-Madison, Madison, Wisconsin, United States of America; University of Texas, UNITED STATES

## Abstract

The evolution of cellulose degradation was a defining event in the history of life. Without efficient decomposition and recycling, dead plant biomass would quickly accumulate and become inaccessible to terrestrial food webs and the global carbon cycle. On land, the primary drivers of plant biomass deconstruction are fungi and bacteria in the soil or associated with herbivorous eukaryotes. While the ecological importance of plant-decomposing microbes is well established, little is known about the distribution or evolution of cellulolytic activity in any bacterial genus. Here we show that in *Streptomyces*, a genus of Actinobacteria abundant in soil and symbiotic niches, the ability to rapidly degrade cellulose is largely restricted to two clades of host-associated strains and is not a conserved characteristic of the *Streptomyces* genus or host-associated strains. Our comparative genomics identify that while plant biomass degrading genes (CAZy) are widespread in *Streptomyces*, key enzyme families are enriched in highly cellulolytic strains. Transcriptomic analyses demonstrate that cellulolytic strains express a suite of multi-domain CAZy enzymes that are coregulated by the CebR transcriptional regulator. Using targeted gene deletions, we verify the importance of a highly expressed cellulase (GH6 family cellobiohydrolase) and the CebR transcriptional repressor to the cellulolytic phenotype. Evolutionary analyses identify complex genomic modifications that drive plant biomass deconstruction in *Streptomyces*, including acquisition and selective retention of CAZy genes and transcriptional regulators. Our results suggest that host-associated niches have selected some symbiotic *Streptomyces* for increased cellulose degrading activity and that symbiotic bacteria are a rich biochemical and enzymatic resource for biotechnology.

## Abstract 2

Cellulose deconstruction helps shape the global carbon cycle; this study shows that high cellulolytic ability evolved in select lineages of the bacterial genus *Streptomyces* through key changes in gene content and transcriptional regulation.

## Author Summary

Only specific microbes can deconstruct the vast stores of carbon within plant biomass. Studying the distribution, diversity, and evolution of these cellulolytic organisms improves our understanding of the ecological functions of microbes in the environment and their contributions to the global carbon cycle. The bacterial genus *Streptomyces* is abundant in soil, appears to readily form symbiotic associations with eukaryotic hosts, and has long been thought to contribute to plant biomass degradation. Here, we show that the ability to rapidly deconstruct cellulose is surprisingly rare in the genus *Streptomyces* but is enriched in symbiotic stains associated with diverse insect hosts that feed on plant biomass. By using a combination of genomic, transcriptomic, and genetic analyses, we identify key changes in gene content and expression that confer cellulolytic activity. Our results support the idea that a complex interplay of genomic changes, occurring over ancient time scales, shapes the evolution of the ecologically important ability to deconstruct plant biomass.

## Introduction

Cellulose is the principal component of plant cell walls and the most abundant organic compound in terrestrial ecosystems [1]. While cellulose deconstruction is critical to ecosystem functioning and the global carbon cycle, only select lineages of fungi and bacteria have evolved the ability to efficiently degrade this highly recalcitrant substrate [2–7]. These cellulolytic microbes are abundant in the soil and are also commonly associated with herbivorous eukaryotes. While a number of these biomass-degrading microbes have been isolated and characterized with genomic, transcriptomic, and biochemical analyses [8–12], relatively little is known about the evolution of this activity in any bacterial genus. Comparative genomic studies demonstrate that important plant biomass-degrading genes are highly variable across multiple taxonomic levels in both fungi and bacteria, suggesting that ecological niche likely drives evolution of cellulolytic ability [3,4,13,14]. Understanding the distribution and evolution of cellulolytic activity in ecologically important bacteria connects microbial diversity to ecosystem function and the global carbon cycle and also provides a powerful approach to identify enzymes for the production of biofuels and other biocommodities.

Actinobacteria from the genus *Streptomyces* are thought to be significant players in the deconstruction of plant biomass [15–17]. These organisms are one of the most abundant genera in soils and recently have been identified living in symbiotic relationships with a wide variety of eukaryotic hosts [18–20]. *Streptomyces* grow as a filamentous hyphal mass, often in low nutrient environments [16,21,22]. They secrete extracellular enzymes (cellulases and proteases) and diverse secondary metabolites with antimicrobial activities [15,23]. Genomic analyses demonstrate that nearly all *Streptomyces* genomes contain Carbohydrate-Active Enzymes (CAZy) and accessory proteins [3]. Individual cellulolytic strains have been isolated from soil and host-associated niches [24,25], and recent work has characterized the transcriptomic and proteomic response of insect-associated strains when grown on plant biomass [11,26].

While *Streptomyces* have been extensively investigated for their ability to produce antibiotic compounds [21,27,28], relatively little is known about the distribution and evolution of plant biomass deconstruction across this, or any, genus of bacteria. Our comparative genomic, transcriptomic, and phylogenetic analyses demonstrate that a small subset of strains evolved the ability to deconstruct cellulose through modified gene content, gene expression, and enzyme architecture. We show that catalytic enzymes and transcription factors are critical for rapid cellulose degradation using targeted gene deletions. These results demonstrate that complex evolutionary processes, including acquisition and selective retention of multiple genes and transcriptional regulators, drive the evolution of plant biomass deconstruction in bacteria.

## Results and Discussion

### Genus-Wide Cellulolytic Ability

**Fig 1:**
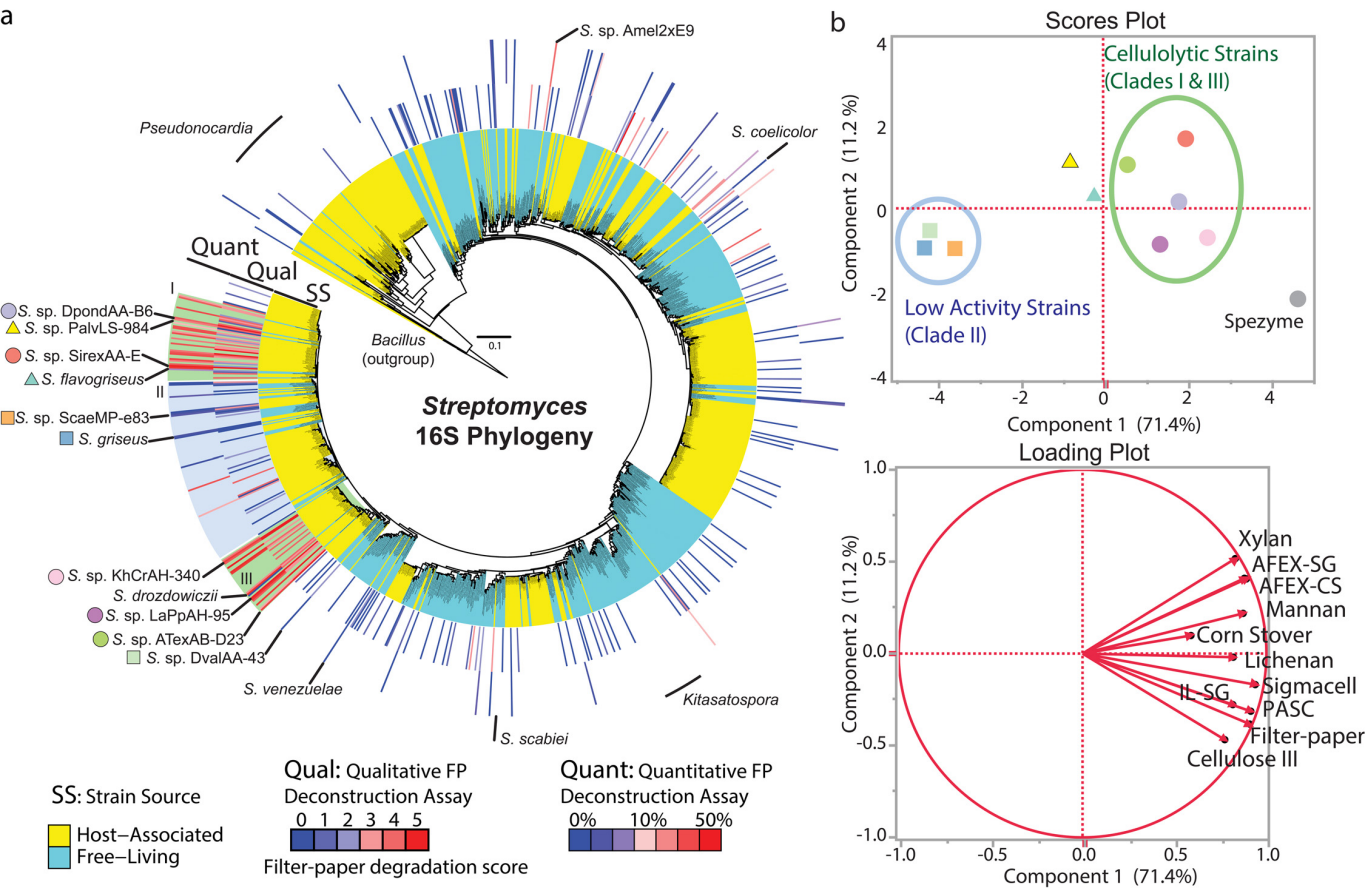
Distribution of cellulolytic ability in the genus *Streptomyces*. **(a)** 16S rRNA gene phylogenetic tree of 1,141 *Streptomyces* strains from free-living (cyan) and host-associated (yellow) environments. The tree is annotated with qualitative cellulose (filter-paper) degradation scores (0: no growth in 3 wk, 5: filter-paper deconstruction in 1 wk) and quantitative cellulose degrading activities (% filter-paper degraded in 10 d). Shading indicates highly cellulolytic clades I and III (green) and related low activity clade II (blue). **(b)** Principle component analysis of cellulose, hemicellulose, and plant biomass degrading activity of *Streptomyces* secretomes. Strains are identified by colored shapes on the tree in panel A. Scores plot shows similarity of polysaccharide degrading activity. Loading plot indicates which substrates influence components 1 and 2 of the scores plot.

We systematically compared the phylogenetic diversity of over 1,100 strains of *Streptomyces* and measured the cellulose degrading activity of 223 diverse strains isolated from free-living (soil and marine) and eukaryotic host-associated niches (Fig 1, S1 Table). We identified strains that exhibited high rates of cellulose degrading activity, deconstructing filter-paper (FP) with comparable activity to the cellulolytic strain *Streptomyces* sp. SirexAA-E [11,20]. Rapid deconstruction of FP was restricted to just 13% of strains tested (29/223, Fig 1), and out of these 29 highly cellulolytic strains, 86% grouped in two phylogenetically distinct clades (I and III). Interestingly, 93% of all strains in these two clades were isolated from taxonomically diverse eukaryotic hosts (e.g., woodwasps, mountain pine beetles, ants, honeybees, and white-rot fungi, Fig 1a). These results suggest that rapid deconstruction of cellulose evolved in these two clades of host-associated strains and is not a conserved characteristic across the *Streptomyces* genus or across all host-associated strains. Rapid cellulose degradation is not limited to clades I and III, as we identify two distantly-related host-associated strains, *Streptomyces* spp. Amel2xE9 and LamerLS-31b, with high cellulolytic activity (Fig 1a). Taken together, these results suggest that the symbiotic environment selects for increased rates of cellulose deconstruction in some lineages of host-associated *Streptomyces*. High cellulolytic activity is not restricted to symbiotic strains, as it has been reported in *Streptomyces reticuli* [29] and several strains isolated from forest soil [24,30]. Future sampling will likely identify additional strains or perhaps even clades with high activity. This cellulose degradation assay identifies strains that are capable of degrading cellulose in pure culture, through the expression and secretion of a full suite of cellulose degrading enzymes. It is likely that some of the low activity strains are capable of deconstructing cellulose in nature; however, in our assay, they are missing critical interactions with environmental community members (e.g., biofilm formation [31], succession of decomposition from fungi [32], mutualistic or parasitic partners) or environmental conditions (e.g., nitrogen limitation, pH, temperature [33,34]).

### Polysaccharide-Degrading Activity of *Streptomyces* Secretomes

To further study the evolution of cellulose degradation in *Streptomyces*, we focused our studies on a subset of the genus consisting of two clades (I and III) that rapidly deconstruct crystalline cellulose, and a low-activity sister clade II (Fig 1). First, we quantified and visualized total secreted proteins produced by representative strains from all three clades when grown with glucose or Ammonia Fiber Expansion (AFEX) pre-treated corn stover as the sole carbon source. The total secreted protein concentration was higher for strains from clades I and III than strains in clade II when grown on AFEX corn stover (S1 Fig). Furthermore, the protein profile produced by strains in clades I and III showed a suite of distinct and abundant polypeptides, while strains in clade II produced a low abundance smear of protein across the lane (S1a Fig). To investigate which components of plant biomass these strains deconstruct, we isolated their secreted proteins and assayed activity on a variety of polysaccharide substrates including cellulose, hemicellulose, and plant biomass (S2 Fig). Principal component analysis of our biochemical assays showed that strains in clades I and III grouped separately from those in clade II (Fig 1b). Some cellulolytic strains from clades I and III had cellulose and biomass degrading activities comparable to a commercial enzyme mixture derived from the secreted proteins of *Trichoderma reesei* Rut-C30 (Spezyme CP, S2 Fig). These results show that some host-associated cellulolytic *Streptomyces* secrete enzymes that efficiently deconstruct crystalline cellulose and plant biomass and thus may contribute to nutrient acquisition of the herbivorous host. Furthermore, these strains may be a promising and largely untapped source of enzymes for the production of biocommodities from plant biomass [35].

### Comparative Genomic Analysis of Plant Biomass Degrading Genes

**Fig 2:**
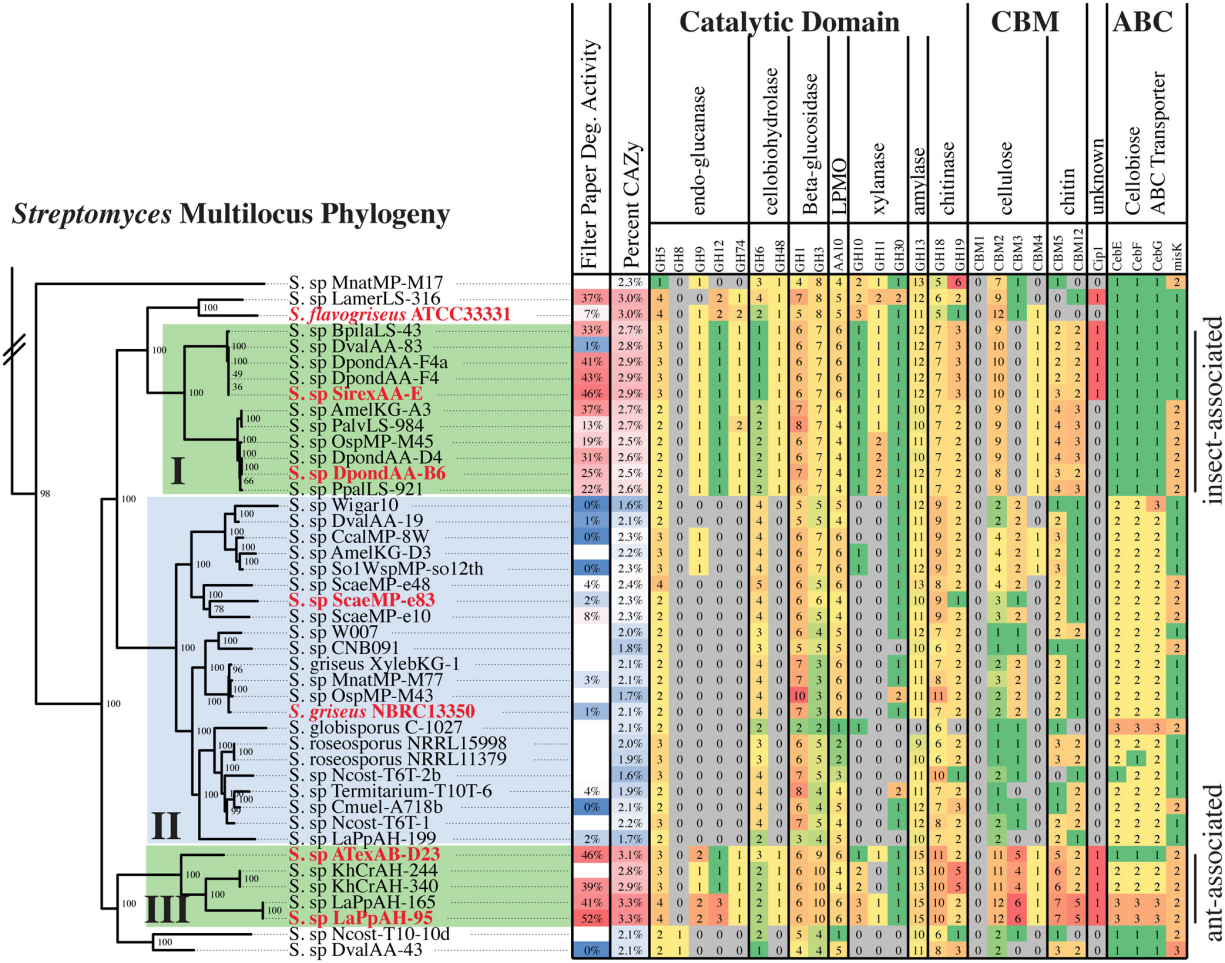
Comparative analysis of CAZy genes across the *Streptomyces* genus. Multilocus phylogenetic tree of clades I–III (full tree: S3 Fig). Taxonomy tree is a RAxML tree calculated from an alignment of 97 genes conserved across all species. Tree is rooted with the outgroup *Kitasatospora setae*, and bootstrap support for each node is indicated (100 bootstraps). Tree is annotated with filter-paper degradation activity and select data from the CAZy and ABC transporter analyses. The number of genes present in each functional category is indicated. Rapid cellulose degrading clades I and III are indicated in green, low activity clade II in blue. Red strains were selected for RNA-seq analysis.

To examine the hypothesis that genomic content is a critical factor determining cellulolytic ability, we sequenced 28 genomes of host-associated *Streptomyces*. These new genomes, combined with previously sequenced genomes, created a dataset containing 67 host-associated and 57 free-living strains, which spans the genus and represents both rapid cellulose degrading strains and low activity strains (S2 Table, Figs 2 and S3). Multilocus phylogenetic analysis shows that clade I is composed of 2 subclades of highly similar strains collected from different hosts and geographies (Fig 2, S1 Table), clade III is composed of three unique species, and clade II contains >15 putative species. Thus, the cellulolytic clades I and III contain relatively few unique species based on genome content but represent a wide variety of host species (e.g., mountain pine beetle, Sirex woodwasp, honey bee, termite, leaf-cutter ants, and arboreal ants), while clade II represents greater species diversity. Additionally, this phylogenetic analysis identified clades of host-associated strains with low cellulolytic activity (S3 Fig). We hypothesize that these strains may contribute a different function to the host, such as defense from pathogens (e.g., *Streptomyces* sp. SPB74 [36]).

Comparative genomic analysis of CAZy content demonstrated that strains across the genus encode the genes necessary to deconstruct plant biomass (Figs 2 and S3). Genes in glycoside hydrolase (GH) families with putative endoglucanase, cellobiohydrolase, β-glucosidase, mannanase, xylanase, chitinase, and lytic polysaccharide monooxygenases (LPMO, AA10) activities were widespread across the genus (Figs 2 and S3). However, highly cellulolytic clades I and III were enriched in total CAZy genes (percent CAZy), in important enzyme families to deconstruct cellulose (GH5, GH6, GH9, GH12, GH48, and AA10) and xylan (GH10, GH11, GH30), and in cellulose-targeting carbohydrate-binding module (CBM) families 2 and 3. Clustering analysis of CAZy profiles demonstrated that cellulolytic clades I and III have similar CAZy gene content, while clade II groups with non-phylogenetically related strains (S4 Fig). Clade II strains all lack a reducing end cellobiohydrolase (GH48) and an endoglucanase (GH9, GH12, GH74), and most strains do not have xylanase genes (GH10, GH11) (Fig 2). The absence of these non-redundant activities likely reduces their ability to deconstruct cellulose and plant biomass. These results suggest an ancient origin for some CAZy genes within this genus followed by selective expansion of specific families in the cellulolytic host-associated clades. Furthermore, they suggest that free-living *Streptomyces* in the soil have not evolved the capacity to rapidly utilize all the components of plant biomass. Interestingly, the CAZy gene content of phytopathogenic strains *Streptomyces scabiei* and *Streptomyces acidiscabies* clusters closely with the cellulolytic strains in clades I and III, suggesting that cellulolytic activity contributes to phytopathogenesis.

Transport of plant biomass breakdown products, free sugars and small oligosaccharides, into the cell is critical for utilization of these recalcitrant carbon sources [37,38]. We analyzed all strains in our genomic dataset for ABC sugar transporter genes (Figs 2, S3, and S5 and S1 Dataset). The cellobiose binding and permease proteins cebE, cebF, and cebG as well as the MsiK ATPase were conserved across all strains in clades I–III and nearly all strains in the genus. Similarly, xylose, N-acetylglucosamine, and multiple-sugar transporters were conserved across most of the genus (S5 Fig). Together, the genomic analyses show variability of the plant biomass deconstructing enzymes across the genus and high conservation of the transporters. Thus, the ability to rapidly deconstruct crystalline cellulose and hemicellulose is not widespread in the *Streptomyces*, whereas utilization of their breakdown products is common to the genus and likely a major carbon source for free-living strains. These “free-living” strains live in complex multi-species communities, where they could cooperate with others microbes to deconstruct plant biomass, or they might specialize in competing for the breakdown products released by the cellulolytic enzymes produced by other microbes.

### Comparative Gene Expression Analysis

**Fig 3:**
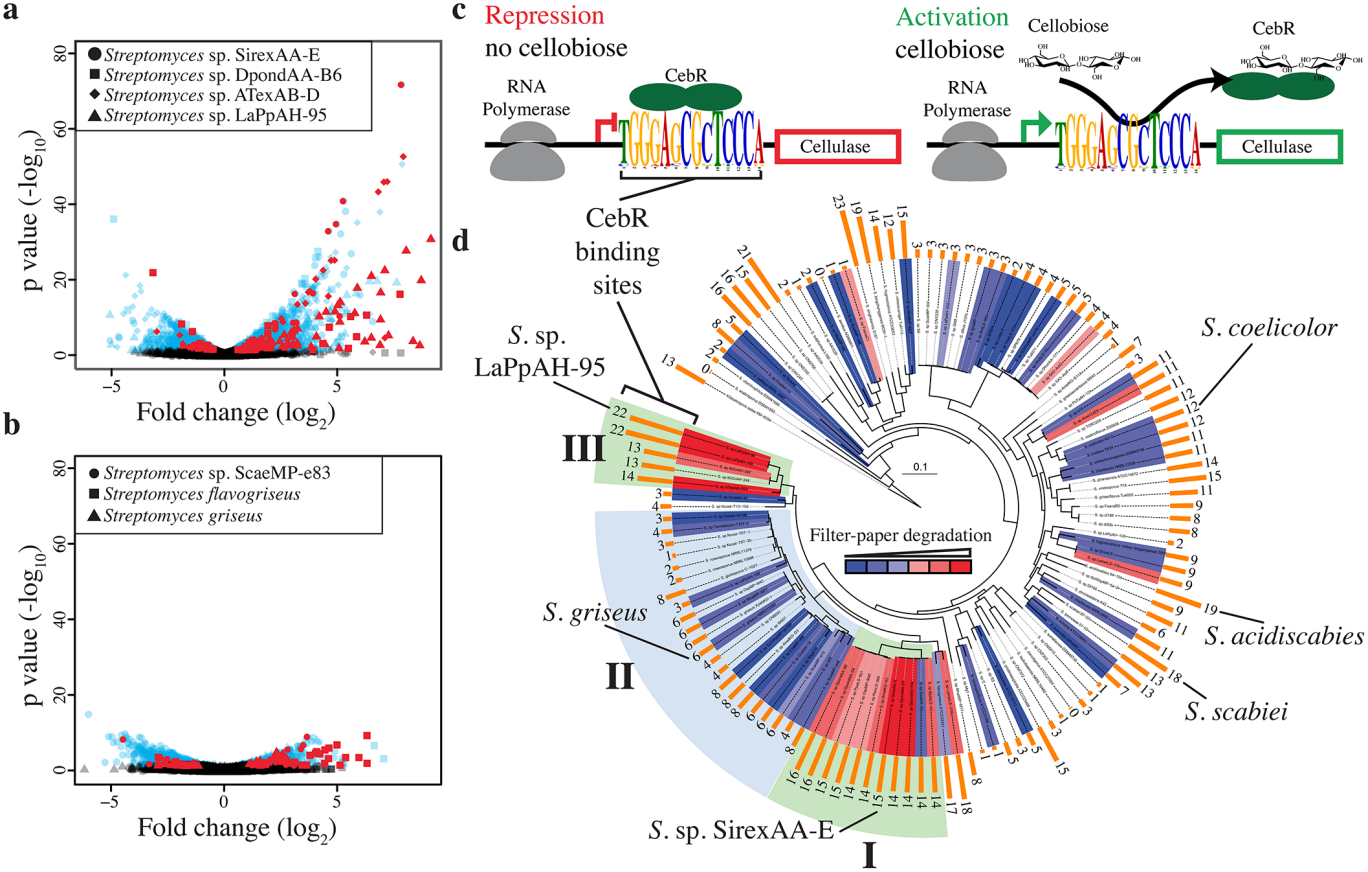
Differential expression and coregulation of biomass degrading genes. Differential expression (DE) of genes from clades I and III **(a)** and clade II **(b)**. Strains were grown with glucose or AFEX pretreated corn stover as the sole carbon source. Each point represents a gene; the shape indicates the strain. The *x*-axis shows the fold change between carbon sources, and the *y*-axis shows the statistical support of the fold change. Black points indicate non-significant DE (*p*-value > 0.05). Red points identify CAZy annotated genes with significant DE. **(c)** Model for negative transcriptional control of CAZy genes by the CebR transcriptional regulator. The CebR binding sequence is the consensus of the top 25 coexpressed genes from each of the four cellulolytic strains. **(d)** *Streptomyces* multilocus phylogenetic tree annotated with quantitative cellulose (filter-paper) degrading activity (red and blue heatmap) and the number of CebR transcriptional regulator binding sites (TGGGAGCGCTCCCA) in the genome (orange bars).

RNA-seq analysis of seven *Streptomyces* from clades I, II, and III (Fig 2, red labels) identified a dramatic disparity in differential gene expression between cellulolytic and non-cellulolytic strains when grown with plant biomass or glucose as the carbon source (S2 Dataset). When grown on AFEX corn stover, the cellulolytic strains SirexAA-E, DpondAA-B6, ATexAB-D23, and LaPpAH-95 induced 23, 16, 49, and 39 genes greater than 4-fold, respectively (Fig 3a), while the clade II strains *Streptomyces griseus* and ScaeMP-e83 induced only two and seven genes greater than 4-fold, respectively, compared to growth on glucose (Fig 3b). CAZy genes represented a large percentage of the highly induced genes in cellulolytic strains (22%–48%), while *S*. *griseus* and ScaeMP-e83 did not induce any CAZy genes greater than 4-fold. When we consider the genes significantly upregulated 2-fold or greater, these patterns are similar. DpondAA-B6, SirexAA-E, ATexAB-D23, and LaPpAH-95 induced nine, 12, 15, and 32 CAZy genes, respectively, including at least six cellulases and two xylanases (S3 Table, S2 Dataset). *S*. *griseus* and ScaeMP-e83 upregulated four and 12 CAZy genes, respectively, greater than 2-fold, including only one cellulase gene, coding for a GH6, and no xylanase genes. Additionally, all strains induced ABC transporter genes, including the cellooligosaccharide sugar binding protein, cebE, indicating that all the strains had the capability to uptake cellulose breakdown products (S3 Table, S2 Dataset). Finally, all strains, except DpondAA-B6, induced expression of multiple transcriptional regulators greater than 2-fold when grown on AFEX corn stover (S3 Table, S2 Dataset).

### Coordinated Expression of CAZy Genes Controlled by the CebR Transcriptional Regulator

The striking contrast in gene expression between rapid cellulose-degrading strains and low activity strains led us to hypothesize that coordinated expression of CAZy genes contributed to the difference in cellulolytic ability. We analyzed the promoter regions of the top 25 induced genes in each RNA-seq dataset and identified a statistically significant 14 bp palindromic sequence (TGGGAGCGCTCCCA, *e*-value 9.2e^-162^) present in the cellulolytic strains that matches the binding element for the CebR transcriptional regulator (S6a Fig). In Actinobacteria, CebR proteins are LacI/GalR-like transcription factors that control gene expression in response to the presence of cellobiose or other small cello-oligosaccharides (Fig 3c) [39–42]. Genus-wide, there was a positive correlation between the number of CebR binding sites in a genome and the cellulolytic ability of the strain (Figs 3d and S6b). We analyzed the genomes of the RNA-seq strains for genes predicted to be directly controlled by CebR and mapped on experimentally observed differential expression values (S4 Table). Interestingly, strains from clades I and III had more genes predicted to be CebR controlled, and a higher percentage of these genes were significantly differentially expressed greater than 2-fold: Clade I strains SirexAA-E had 10/18 CebR controlled genes expressed >2-fold, DpondAA-B6 11/19; Clade III strains ATexAB-D23 14/19, LaPpAH-95 21/30; and Clade II strains *S*. *griseus* 2/8, ScaeMP-e83 3/6. Thus, identification of strains enriched with CebR binding sites likely predicts increased cellulolytic ability. Remarkably, the phytopathogenic strains *S*. *scabiei* and *S*. *acidiscabies* have 18 and 19 CebR sites in their genomes, respectively, including a site in the promoter region of the thaxtomin toxin biosynthetic gene that controls toxin production [43].

**Fig 4:**
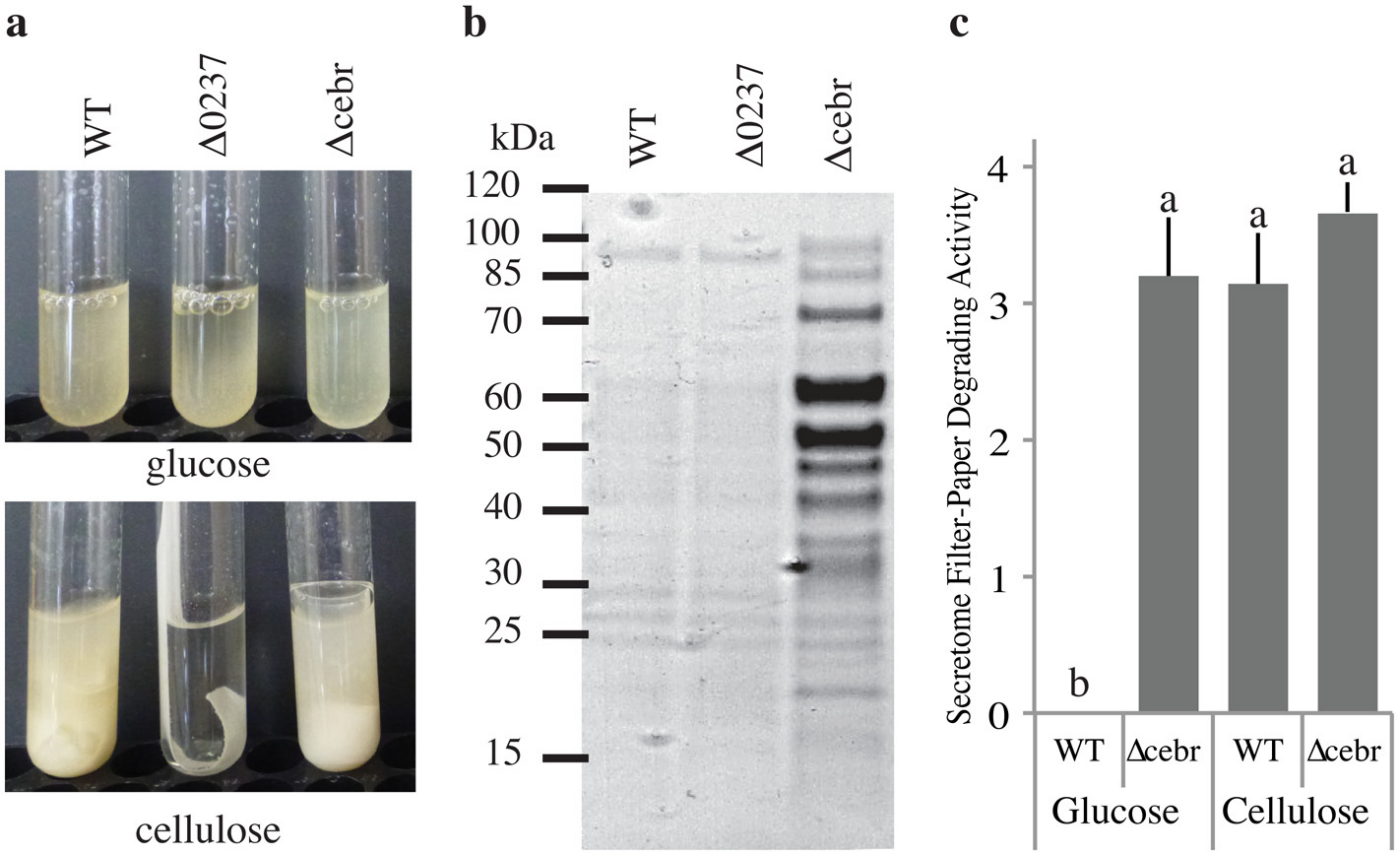
Genetic analysis of *Streptomyces* sp. SirexAA-E. **(a)** Phenotypic analysis of *Streptomyces* sp. SirexAA-E wild-type (wt), Δ0237 (GH6), and Δcebr (cellulase transcriptional regulator) strains grown on glucose or cellulose as the sole carbon source. **(b)** Secreted protein profile of wt, Δ0237, and Δcebr strains grown in glucose media. **(c)** Filter-paper degrading activity (mg glucose released per mg total protein) of secreted proteins isolated from wt and Δcebr strains grown with glucose or cellulose as the carbon source. Statistically significant differences are indicated.

We confirmed that CebR controls coordinated expression of CAZy genes by generating a CebR deletion strain in SirexAA-E (SACTE_2285, Δcebr, Fig 4). This strain constitutively expressed and secreted a suite of cellulolytic enzymes in cellulose-free media (Figs 4b–4c and S7). Constitutive expression of a transgenic CebR in the Δcebr strain resulted in wild-type levels of secreted cellulases in glucose media and reduced protein when grown in cellulose media (S8b Fig).

Comparison of a phylogenetic tree of the CebR repressor with the *Streptomyces* phylogeny shows incongruence of this transcription factor with the evolutionary history of the strains (S9a Fig). Cellulolytic strains with a large number of CebR binding sites in their genomes contained more similar CebR gene sequences than the related species with low activity. Molecular evolutionary analysis showed that CebR from the low activity strains contained only residues with neutral (1.2 > ω > 1) or purifying selection (ω < 1.2, S9b Fig), while residues in the cellulolytic clades showed positive selection to diversify (ω > 1.2). Sites with positive selection were located on the surface of the protein and may influence ligand or DNA binding directly or through allosteric interactions. Together, the gene expression and molecular evolution data suggest an important connection between the evolution of the CebR transcriptional regulator, the number of CebR binding sequences in the genome, and the cellulolytic ability of the strain.

### Analysis of Important Enzyme Families

In three of the four cellulolytic strains (SirexAA-E, ATexAB-D23, and LaPpAH-95), a GH6 family non-reducing end cellobiohydrolase was the most highly induced gene when grown on AFEX corn stover and was significantly induced greater than 2-fold in all strains (S2 Dataset). To test the importance of the GH6 enzyme to the cellulolytic phenotype, we generated an in-frame deletion of the GH6 gene (SACTE_0237) in the highly cellulolytic strain SirexAA-E (Figs 4a and S7). The mutant strain (Δ0237) grew normally in glucose media; however, no growth, secreted proteins, or significant cellulolytic activity was observed in cellulose media (S7 Fig). To confirm that this phenotype was due solely to the loss of the GH6 protein, we generated a Δ0237 ΔcebR double mutant (S7 Fig) to constitutively express the cellulases. We observed that the secretome was nearly identical to the ΔcebR single mutant except for the missing GH6 protein band (S7e Fig). The filter-paper degrading activity of the double mutant was reduced to near zero, similar to the single Δ0237 mutant (S7d Fig). The mutant phenotype was rescued by integration of a constitutively expressed transgene (ermE*::SACTE_0237) into the Δ0237 strain (S8b Fig). These results support a growing body of literature that suggest the importance of individual CAZy genes for cellulolytic ability [44]. Furthermore, they demonstrate that rapid cellulolytic activity is the product of a complete suite of enzymes (endoglucanase, reducing and non-reducing end cellobiohydrolase, LPMO), and absence of any one of these enzymes results in limited activity. Thus, the absence of the endoglucanase genes (GH9, GH12, GH74), a reducing end cellobiohydrolase gene (GH48), and xylanase genes (GH10 and GH11) in most clade II strains likely has a major impact on their cellulose and plant biomass degrading ability in isolation.

The importance of lytic polysaccharide monooxygenases (LPMOs, AA10) to the deconstruction of recalcitrant polysaccharides is increasingly recognized [45–48]. LPMOs are abundant in *Streptomyces* genomes and include both chitinolytic and cellulolytic AA10 genes [49]. Our genus-wide genomic analysis shows that AA10 genes are present in all strains, and there is little difference in the number of genes between highly cellulolytic clades I and III and low activity clade II (Fig 2). To gain further insight into their variation and utilization, we examined the phylogenetic distribution of AA10 proteins from clades I–III mapped with RNA-seq gene expression data and transcriptional regulator binding sites (CebR, S10 Fig). This tree shows that strains in clade I and III have three homologs in the cellulolytic clade and three homologs in the chitinolytic clade, while clade II strains (e.g., *S*. *griseus*) have two cellulolytic AA10s and four chitinolytic AA10s. The most highly expressed homolog (clade A1, S10 Fig) was conserved in strains from all clades. All protein sequences in clade A1 contained a CBM2 domain, and analysis of the upstream DNA identified a CebR transcription factor binding site. However, differential expression of the *S*. *griseus* gene was less than half of those from strains in clades I and III (SirexAA-E, DpondAA-B6, LaPpAH-95, and ATexAB-D23) (S10 Fig and S2 Dataset). The second most highly expressed homolog (clade A2, S10 Fig) only contained sequences from the rapid cellulose-degrading clade I and III strains. These sequences were single domain proteins, and the upstream genomic DNA encodes a CebR binding site. Most genes in the chitinolytic clade had low and insignificant differential expression values when grown on AFEX corn stover, suggesting a different regulatory system. Together, these results suggest small but potentially significant differences in AA10 homologs between rapid crystalline cellulose degrading strains and strains with low activity.

Interestingly, three cellulolytic strains (SirexAA-E, ATexAB-D23, and LaPpAH-95) strongly induced expression of a homolog of Cip1 from *T*. *reesei* (42.2% identity). Cip1 is highly expressed in *T*. *reesei* during growth on cellulose, but the biochemical function is unknown [50]. Cip1 homologs are present in only 3 bacterial phyla (Acintobacteria, Proteobacteria, and Chloroflexi) and present in just 21% of *Streptomyces* genomes in this dataset (S2 and S11 Figs). Phylogenetic analysis of Cip1 protein sequences identifies monophyletic clades for the Ascomycota, Actinobacteria, and Proteobacteria phyla (S11 Fig). *Streptomyces* Cip1 sequences are polyphyletic and incongruent with the multilocus phylogeny (S3 and S11 Figs). These data suggest possible lateral transfer of Cip1 within the *Streptomyces* genus. Additionally, the four cellulolytic strains upregulated a combined 20 genes (greater than 4-fold) annotated as unknown function, suggesting that these *Streptomyces* may contain additional proteins with novel biochemistry.

### Evolution of CAZy Genes in Cellulolytic Strains

**Fig 5:**
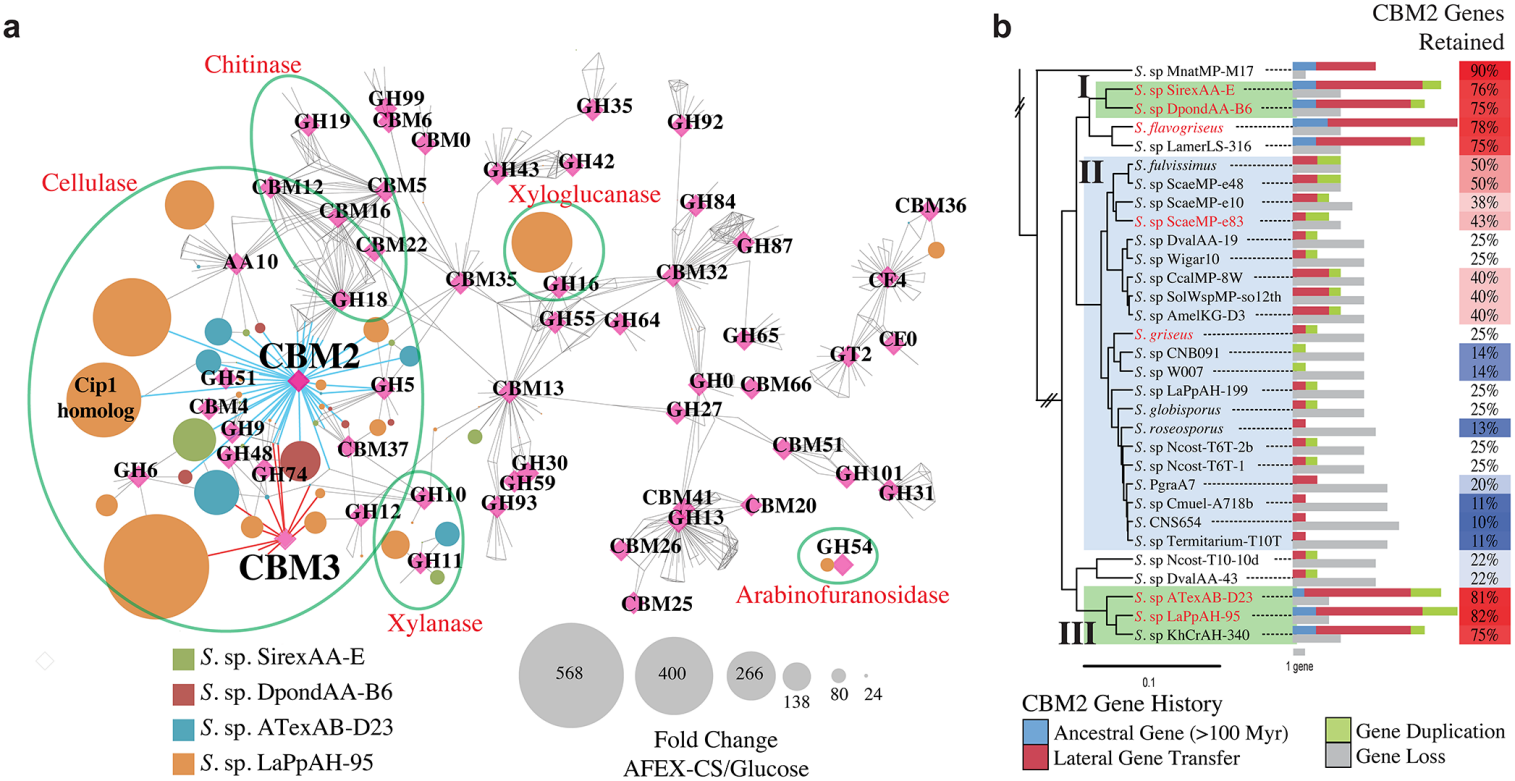
Multi-domain enzyme expression and evolution. **(a)** Protein similarity network of CAZymes present in four cellulolytic *Streptomyces* strains. Nodes are proteins (circles) or CAZy functional categories (magenta diamonds); edges indicate that the gene belongs to the respective CAZy family or BLAST similarity with an *e*-value < 1xe^-50^. Node size represents the fold-change in RNA abundance between glucose and AFEX corn stover grown cells. CBM2 and CBM3 linked proteins are indicated with blue and red edges, respectively. **(b)** Evolutionary expansion of CBM2 domains in cellulolytic *Streptomyces*. CBM2 domain gain and loss events for strains in cellulolytic clades I & III (green shading) and clade II (blue shading) mapped onto the multilocus phylogenetic tree. CBM2 retention rates (genes retained / total possible genes) are identified by the heatmap.

Our comparative genomic and transcriptomic analyses also identified differences in domain architecture of cellulolytic enzymes, which is critical for substrate specificity and specific activity [9,51]. Clade I is enriched in CBM2 domains, while clade III is enriched in both CBM2 and CBM3 domains (Fig 2). Additionally, a protein homology network showed that nearly all highly expressed CAZy enzymes contained both a catalytic and a CBM domain (Fig 5a). We hypothesized that expansion of multi-domain proteins in cellulolytic strains contributes to their biomass degrading ability. To test this hypothesis, we first modeled the evolutionary history of each CAZy gene family in *Streptomyces* including gene origin (lateral gene transfer, gene duplication, or ancestral) and loss events (S12 Fig). Although the CBM2 genes are the most abundant family in extant genomes, only 55% of total possible genes were retained across the evolutionary history of the genus. When gain and loss of CBM2 genes were plotted on individual species, we observed a dramatic difference between the rapid crystalline cellulose degrading strains and strains with low activity (Figs 5b and S13). Clades I and III were enriched in the total number of CBM2 domains and had higher retention rates than clade II (79% versus 27% retained on average, respectively). The largest inferred source of CBM2 genes in cellulolytic clades was horizontal gene transfer, followed by gene duplication, and then vertical inheritance (ancestral genes, Fig 5b). Interestingly, CBM3 genes were expanded and retained almost exclusively in clade III (S13 Fig). In contrast, the GH6 family had a lower retention rate in the highly cellulolytic clades than the low activity clade, 40% and 53%, respectively (S13 Fig). These results indicate that while the CBM2 and CBM3 families of genes are under selection to expand and diversify in the cellulolytic strains, there is no selective advantage for additional GH6 family genes in these strains. Together, these data suggest that targeted expansion of the CBM2 and CBM3 families through horizontal transfer and selective gene retention likely contributed to the evolution of cellulolytic ability in host-associated *Streptomyces*. Furthermore, they emphasize that clade II strains have not been selected for increased ability to bind and deconstruct crystalline cellulose, and thus these strains may obtain sufficient carbon from plant biomass breakdown products.

Our genomic, proteomic, and transcriptomic analyses of *Streptomyces* identify key genome modifications that increase cellulolytic ability, and we determine the evolutionary processes that contributed to these changes. While genomic content predicts that nearly all *Streptomyces* are capable of crystalline cellulose deconstruction, our results demonstrate that high activity is limited to select lineages of host-associated strains. Selective pressure from the symbiotic environment likely selects these strains for increased CAZy gene content, retention of horizontally transferred genes, and expansion of regulatory elements. In contrast, free-living soil strains, having evolved within diverse communities, likely have experienced limited selection for rapid cellulose degrading activity in isolation. Evolution-focused multiomic studies of cellulolytic microbes are critical for understanding the functional contributions of environmental microbes to the carbon cycle, ecosystem processes, and climate change, as well as providing high-interest organisms and enzymes for the biofuels industry.

## Materials and Methods

### Isolation of *Streptomyces*

Free-living strains were obtained from the USDA ARS Culture Collection at the National Center for Agricultural Utilization Research in Peoria, Illinois. Host-associated strains were isolated by the Currie-lab from diverse insects, plants, and fungi (S1 Table). In all cases, hosts were surface sterilized in 95% ethanol for 1 min, rinsed twice in sterile phosphate-buffered saline (PBS), homogenized in PBS, and dilution plated onto Chitin minimal medium [25]. Colony forming units were isolated and maintained on ISP2 (Difco).

### Culturing of Organisms

Strains were grown in M63 minimal medium at pH 6.8 [11, 52]. Qualitative filter-paper deconstructive assays were performed with triplicate test tubes containing 5 mL M63 with either 0.5% w/v glucose or a 1 x 10 cm strip of Whatman #1 filter-paper (Millipore, Billerica, MA) as the sole carbon source. Tubes were observed over 3 wk and scored: 0 = no growth in 3 wk; 1 = growth in 2–3 wk, no degradation; 2 = growth in 1 wk, no degradation; 3 = degradation in 2–3 weeks; 4 = low degradation in 1 wk and heavy degradation in 2 wk; 5 = heavy degradation in 1 wk.

Large-scale growth was performed in flasks containing 50 mL M63 with either 0.5% w/v glucose or sterile AFEX-pretreated corn stover (GLBRC Corn Stover UW 2009 AFEX, AFEX ID 712–192) and was collected after 1 wk and used for enzyme assays and transcriptomics. All cultures were incubated at 30°C with shaking at 250 rpm.

### Quantification of Residual Cellulose

Cellulose degradation was quantified using previously described methods [26]. At least three replicates were performed for each strain.

### Analysis of Secreted Enzymes

Supernatants were prepared from growing cultures by first centrifugation, then filtration, and enzyme activity was measured using dinitrosalicylic acid (DNS) assays [11,26,53]. Reducing sugar assays were performed against Whatman #1 filter-paper (GE Healthcare Life Sciences, Pittsburgh, PA), Sigmacell-20 (Sigma-Aldrich, St. Louis, MO), cellulose-III (a generous gift from Dr. Dale at Michigan State University, East Lansing [54]), phosphoric acid swollen cellulose (PASC) [55], birch-wood xylan (Sigma-Aldrich, St. Louis, MO), D-mannan (Megazyme, Ireland), corn stover (GLBRC 2009 Corn Stover), AFEX-pretreated corn stover (GLBRC Corn Stover UW 2009 AFEX, AFEX ID 712–192), and ionic-liquid pretreated switchgrass (a generous gift from Department of Energy Joint Bioenergy Institute, CA). The industrial enzyme cocktail, Spezyme CP (Genencor, Palo Alto, CA), was used as a comparison. Statistical analyses were performed in JMP Pro (SAS, Cary, NC).

For SDS-PAGE analysis, strains were gown with either glucose or AFEX-corn stover as the sole carbon source for 7 d and harvested by centrifugation and filtration. Total protein in each secretome was measured with a Bio-Rad protein assay (Bio-Rad, Hercules, CA), and BSA was used to generate a standard curve. Secretomes were normalized to equal protein concentrations and separated on a 4%–20% Bio-Rad Criteron gel. Gel was stained with Bio-Rad Bio-Safe coomassie stain.

### Genomic Sequencing and Phylogenetic Analysis

16S rRNA gene sequences for *Streptomyces* and representative Actinobacteria were downloaded from NCBI refseq. These were combined with sequences from strains isolated by the Currie-lab [25] and sequences extracted from *Streptomyces* genome assemblies. The full set of sequences was aligned using MAFFT [56] and trimmed based on visual inspection. The alignment was used to generate a phylogeny using RAxML-7.2.6. with the GTRGAMMA substitution model [57].

Genome sequences for 28 host-associated *Streptomyces* were generated (S2 Table) at the Joint Genome Institute (JGI [11,20]). Library construction and sequencing methods used at the JGI can be found at http://www.jgi.doe.gov/. Genome annotations were generated at the JGI. Putative CAZy gene annotations were generated using a custom script [11]. These 28 genomes were combined with 99 other high-quality *Streptomyces* genomes to create a dataset of 127 genomes. Multilocus phylogenetic analysis was performed as described in Book et al. 2014 [26] using 97 conserved genes families that were found to be present in all 127 genomes. CAZy gene profile similarity tree was generated by calculating a similarity matrix with the Spearman algorithm and then generating a neighbor-joining tree with phylip. ABC sugar transporters were identified by GhostKOALA analysis of all 127 *Streptomyces* genomes [58]. Genes annotated as ABC sugar transporters as defined by standard KEGG ontology were parsed and collected in S1 Dataset.

CebR phylogenetic trees were constructed as described in Book et al. 2014 [49]. Briefly, CebR homolog protein sequences were identified by BLAST analysis of NCBI and sequences were aligned with MUSCLE [59]. The phylogenetic tree was generated using the MrBayes [60] code on CIPRES Science Gateway with a calculated standard deviation of ≤ 0.05. Non-default parameters were set to mcmc, ngen = 10,000,000, temp = 0.200, burninfrac = 0.25, stoprule = No, sump burnin = 4,000, and sumt burnin = 4,000. Phylogenetic analysis of Cip1 and AA10 were generated with similar methods. The Cip1 dataset was collected with a NCBI refseq database BLAST search with an *e*-value cutoff of 1e^-10^. The AA10 data set was generated by collecting all AA10 annotated genes from the above CAZy analysis in Clade I-III strains.

### RNA Analysis

RNA was purified from *Streptomyces* strains using a modified phenol-chloroform extraction protocol, DNase treatment, and ribosomal RNA removal [11,26,61]. cDNA library construction and single-end 100 bp Illumina HiSeq2000 sequencing was performed at the University of Wisconsin Biotechnology Center (Madison, WI). Data were demultiplexed using the CASAVA software package, and analysis was performed using a previously published pipeline [26]. BLAST comparison of all CAZy annotated genes in *Streptomyces* strains SirexAA-E, DpondAA-B6, LaPpAH-95, and ATexAB-D23 were used to make homology networks in Cytoscape [62]. Proteins (circles) with a BLAST *e*-value < 1e^-50^ were connected with an edge, as were CAZy family annotations (triangles).

### CebR Analysis

Promoter sequence-based motifs were identified using MEME [63]. We analyzed DNA sequences 500 bp upstream of the start codon for the top 25 most differently expressed genes in each RNA-seq dataset. The occurrence of motifs in the sequence was assumed to be distributed zero to three per sequence. The consensus motif was generated by collecting a dataset that combines promoter sequences (500 bp upstream of start) from *Streptomyces* spp. SirexAA-E, DpondAA-B6, LaPpAH-95, *flavogriseus*, and ATexAB-D23. This dataset of 125 sequences was then analyzed with MEME, and the consensus motif and position weight matrix were identified. CebR binding site analysis was performed using the FIMO analysis in the MEME suite using the CebR consensus motif generated by MEME and searched against all *Streptomyces* genomes in our dataset. Results were filtered to an *e*-value cutoff of 1e^-7^ [64].

Evolutionary rate estimation was done as described in Book et al. 2014 [49]. DNA coding sequences for CebR genes were collected from NCBI and codon alignments were generated with MUSCLE. Codon alignments were masked with Zorro to generate quality scores for codon positions [65], and then a phylogenetic tree was generated with RAxML using the masking scores [57]. Site-specific codon substitution models were generated using the CODEML program in PAML [66], with model = 0, NSsites = 3, ncatG = 3, fix_kappa = 0, fix_omega = 0, cleandata = 1, and fix_blength = 2. Protein homology models were generated by aligning proteins sequences to the 1BDH structure with ITASSER [67].

### Lateral Gene Transfer Analysis

The AnGST algorithm [68] was used to infer horizontal gene transfer, gene duplication, and gene loss of CAZy domains across the *Streptomyces* phylogeny. The protein sequences for dbCAN annotated domain families were aligned using MAFFT [56]. These alignments were then reverse translated to codon alignments, RAxML-7.2.6 [57] was used to generate 10 bootstrapped alignments, and FastTree 2.1.7 [69] was used to generate phylogenies from these bootstrap alignments. The 10 bootstrap phylogenies were then used as the input for AnGST with default parameters. A molecular clock-based species tree of *Streptomyces* genomes, plus an additional 20 Actinobacterial genomes used as outgroups, was generated using Reltime [70] with Cyanobacterial fossils and the *Salmonella*/*Escherichia coli* divergence time used as calibration points.

### *Streptomyces* sp. SirexAA-E Genetics

*E*. *coli* XL1 Blue MR (Stratagene, La Jolla, CA) and *E*. *coli* DH10b (Life Technologies, Carlsbad, CA) were used for routine subcloning, plasmid preparations, and cosmid library preparations. *E*. *coli* S17-1 [16] was the donor strain for intergeneric conjugation. The pGEM series of vectors (Promega, Madison, WI) and Litmus 28 and 38 (New England Biolabs, Beverly, MA) were from commercial sources. pANT841 [71], pOJ260 [72], and pSET152 [72] were described previously. The ermE* promoter was obtained from pWHM1250 [73]. SuperCos1 (Stratagene) was used for the cosmid library. DNA isolations and manipulations in *E*. *coli* [71] and *Streptomyces* [16] were carried out according to standard procedures.

A cosmid library of *Streptomyces* sp. SirexAA-E chromosomal DNA was constructed by partially digesting DNA with *Sau3AI*, dephosphorylating, and ligating into the *BamHI* site of SuperCos1. The ligation mixture was packaged using Gigapak III XL packaging mix (Agilent Technologies, Santa Clara, CA) and transduced into *E*. *coli* XLI Blue MR cells. The library was screened by colony hybridizations using digoxigenin-labeled PCR products of the CebR gene and the GH6 gene to find cosmids containing these genes within the center portion of the inserts. Cosmids were end sequenced to determine their exact content. A ~7.5 kb *KpnI* fragment of DNA containing the GH6 (SACTE_0237) gene was subcloned from the cosmid library into pGEM7Zf. A ~5 kb *PstI-HindIII* fragment was transferred into Litmus38, and the resulting plasmid was cut with *SacI* and religated, removing 942 bp, thereby creating an in-frame deletion within the SACTE_0237 gene. The entire insert was then transferred into pOJ260, yielding pOJ260Δ0237. This construct was transformed into *E*. *coli* S17-1 in preparation for intergeneric conjugation.

To generate the CebR deletion, a ~9 kb XhoI fragment was isolated from the cosmid library and cloned into a standard cloning vector. From this, a 2.1 kb *SphI-PshAI* fragment and a 2 kb *MluI*(Klenow treated)-*BamHI* fragment were isolated. Both fragments were ligated into pANT841, thereby creating a ~4 kb insert with an 810 bp in-frame deletion in the CebR gene. The entire region was removed as an *EcoRI-HindIII* fragment and cloned into pOJ260, yielding pOJ260Δcebr. This construct was transformed into *E*. *coli* S17-1 in preparation for intergeneric conjugation.

*E*. *coli–Streptomyces* intergeneric conjugation was carried out as described in Galm et al. [74] using standard literature conditions [16] with minor modifications. Exconjugants were picked to fresh supplemented ISP4 plates containing naladixic acid and apramycin. Colonies that grew were screened via PCR to ensure they had both the WT and the mutant copies of the gene in the chromosome. These “single crossover” mutants were then grown for several rounds on nonselective media and tested again by PCR for the loss of the WT copy of the gene. Those mutants showing only the deleted copy of the gene were then confirmed via Southern hybridization. Southern analysis and colony lifts, digoxigenin labeling of DNA probes, blotting to Hybond-N+, hybridization, and detection were performed according to the protocols provided by the manufacturers (GE Healthcare, Piscataway, NJ and Roche Diagnostics Corp., Indianapolis, IN).

Mutations were rescued by inserting a WT copy of each gene of interest behind the constitutive ermE* promoter into the site-specific integrating vector pSET152 and introducing this plasmid into the respective mutant strains via intergeneric conjugation using *E*. *coli S17-1* as the donor strain. Exconjugants were picked to fresh supplemented ISP4 plates containing naladixic acid and apramycin. Colonies that grew were screened via PCR to ensure they had the WT copy of the gene present behind the ermE* promoter. Empty pSET152 was used as a control.

Protein profiles were obtained from strains of interest by growing 5 ml starter cultures in R2YE for 2–3 d. A 100 ul aliquot of these cultures was then used to inoculate 5 ml of M63 media supplemented with either glucose or cellulosic filter-paper as the sole carbon source. Cultures were grown in a 30°C shaking incubator overnight and aliquots were centrifuged, run on a 4%–20% Criterion TGX Gel, and visualized using Coomassie Stain (Bio-Rad, Hercules, CA). Reducing sugar assays were performed as described above with filter-paper as the carbon source and a 30°C incubation for 20 h.

## Supporting Information

S1 Dataset Comparative genomic analysis of all ABC sugar transporters in *Streptomyces*. Provided as an Excel spreadsheet.
(XLSX)

S2 Dataset RNA-seq differential expression data table for SirexAA-E, DpondAA-B6, ATexAB-D23, LaPpAH-95, *S*. *griseus*, and ScaeMP-e83. Provided as an Excel spreadsheet.
(XLSX)

S1 Fig Secreted protein profiles of *Streptomyces* strains from clades I–III. **(a)** SDS-PAGE of total secreted proteins from each strain grown on either glucose (glu) or AFEX corn stover (CS) as the sole carbon source. Phylogenetic relationship of strains indicated by cladogram. **(b)** Total secreted protein concentration of strains grown with glucose or AFEX-CS as the carbon source.
(EPS)

S2 Fig Polysaccharide degrading enzyme activities of *Streptomyces* secretomes. **(a)** Cellulose degrading activity of secretomes isolated from cellulolytic and clade II strains using filter-paper, Sigmacell (Avicel), Cellulose III, and Phosphoric acid swollen cellulose (PASC). **(b)** Hemicellulose degrading activity with xylan, mannan, and lichenan substrates. **(c)** Plant biomass degrading activity of untreated and pre-treated substrates; corn stover (untreated), AFEX pre-treated corn stover (AFEX-CS) and switchgrass (AFEX-SG), and ionic liquid pretreated switchgrass (IL-SG).
(EPS)

S3 Fig Comparative analysis of CAZy genes across the *Streptomyces* genus. Multilocus phylogenetic tree of 126 strains with genomes. Taxonomy tree is a RAxML tree calculated from an alignment of 97 genes conserved across all species. Tree is rooted with the outgroup *Kitasatospora setae*, and bootstrap support for each node is indicated (100 bootstraps). Tree is annotated with filter-paper degradation activity and select data from the CAZy and ABC transporter analyses. Numbers in the heatmap indicate the number of genes present in each functional category. Cellulolytic clades I and III are indicated in green, low activity clade II in blue. Yellow strains are phytopathogens.
(EPS)

S4 Fig Comparative analysis of *Streptomyces* phylogeny and the CAZy gene profile. *Streptomyces* multilocus phylogeny is shown on the left side and is annotated with the filter-paper degrading activity. The tree on the right represents the similarity of CAZy gene content in each genome. Total CAZy profiles were analyzed by Spearman rank correlation, and the tree was generated by phylip. Highly cellulolytic clades I and III are highlighted in green, low activity clade II in blue, and phytopathogens in yellow.
(EPS)

S5 Fig Comparative analysis of ABC sugar transporter genes across the *Streptomyces* genus. Multilocus phylogenetic tree of 126 strains with genomes. Taxonomy tree is a RAxML tree calculated from an alignment of 97 genes conserved across all species. Tree is rooted with the outgroup *Kitasatospora setae*, and bootstrap support for each node is indicated (100 bootstraps). Numbers in the heatmap indicate the number of genes present in each ABC sugar transporter KEGG category. Cellulolytic clades I and III are indicated in green, low activity clade II in blue. Yellow strains are phytopathogens.
(EPS)

S6 Fig Promoter motif analysis of cellulolytic and non-cellulolytic *Streptomyces*. **(a)** Promoter sequences (500 bp upstream of start codon) for the top 25 most highly induced genes identified in the RNA-seq dataset of each strain were analyzed for conserved motifs. The consensus motif was generated by combining the top 25 genes from each strain and running MEME analysis. **(b)** Correlation between the number of predicted CebR binding sites in each genome and the filter-paper degrading activity of each strain. Blue line indicates the linear regression line for the scatter plot, and the R^2^ coefficient of determination is shown.
(EPS)

S7 Fig Genetic engineering of *Streptomyces* sp. SirexAA-E. **(a)** Southern blot analysis of Δ0237 mutants showing deletion of full length SACTE_0237 (GH6-CBM2 enzyme). (**b)** Southern blot analysis of Δcebr mutants showing deletion of full length SACTE_2285 (CebR transcriptional regulator). **(c)** Phenotypic analysis of Δ0237, Δcebr, and Δ0237Δcebr double mutants grown with glucose or filter-paper as the sole carbon source. **(d)** Quantitative filter-paper degrading activity of Δ0237, Δcebr, and Δ0237Δcebr mutants. **(e)** Secreted protein profile of Δ0237, Δcebr, and Δ0237Δcebr mutants grown with glucose as the sole carbon source.
(EPS)

S8 Fig Secreted protein profiles of rescued *Streptomyces* sp. SirexAA-E deletion strains. **(a)** Rescue of WT protein profile of Δ0237 strain with pSET152EE::0237 transgene. This construction incorporates the WT SACTE_0237 into the genome and is driven by the constitutive ermE* promoter. The rescued strain has regained ability to grow and secrete proteins in filter-paper media. Glucose media shows constitutive expression of the protein (SACTE_0237). **(b)** Rescue of WT protein profile of Δcebr strain with pSET152EE::CebR transgene in glucose media.
(EPS)

S9 Fig Evolution of the CebR transcriptional regulator in cellulolytic and low activity strains. **(a)** Comparison of *Streptomyces* multilocus phylogenetic tree and the CebR gene tree. Tree is rooted with the outgroup *Streptomyces* sp. MnaMP-M17. Posterior probabilities for each node are indicated. CebR gene tree is annotated with the number of CebR binding sites (TGGGAGCGCTCCCA) present in each genome. **(b)** Site-specific estimation of dN/dS ratios for the cellulolytic clade I and clade II. Positively selected residues are colored in red, neutral in grey, and negative in blue. The *x*-axis corresponds to the protein sequence, and the *y*-axis corresponds to the posterior probability of the estimation. dN/dS ratios were also mapped onto the modeled structures for SACTE_2285 and SGR_04765. Colors correspond to selection rates described above.
(EPS)

S10 Fig Phylogenetic diversity of AA10 genes from strains in clades I–III. Bayesian phylogenetic tree of AA10 protein homologs. Tree is midpoint rooted, and nodes are labeled with posterior probability scores. Putative cellulolytic and chitinolytic clades are shaded. Tree is annotated with CAZy domains, CebR regulatory sequences, and RNA-seq expression data. The two most highly expressed and induced clades are indicated, A1 and A2, respectively.
(EPS)

S11 Fig Phylogenetic diversity of Cip1 in bacteria and fungi. Bayesian phylogenetic tree of all Cip1 protein homologs in the NCBI refseq database with an *e*-value < 1e^-10^. Fungal and bacterial phyla are colored, as well as the *Streptomyces* genus. Tree is midpoint rooted, and nodes are labeled with posterior probability scores.
(EPS)

S12 Fig Evolution of CAZy gene families in *Streptomyces*. Gene gain and loss events for all CAZy families. Gene loss is shown on the left with grey bars. Extant genes are shown on the right and are separated into three categories: ancestral genes older than 100 my are shown in blue, laterally transferred genes within the last 100 my are shown in red, and duplicated genes are shown in green.
(EPS)

S13 Fig Evolution of CBM2, CBM3, and GH6 gene families. Gene gain and loss events for select gene families in our phylogenomic dataset. Gene loss is shown with grey bars. Gene gain is separated into three categories: ancestral genes older than 100 my are shown in blue, laterally transferred genes within the last 100 my are shown in red, and duplicated genes are shown in green. Cellulolytic clades (I and III) are highlighted in green and clade II in blue.
(EPS)

S1 Table Qualitative filter-paper degrading activity of *Streptomyces* [20,26,75–82]. (XLSX)

S2 Table Genome Accession Numbers. (XLSX)

S3 Table Summary of genes upregulated ≥2-fold. (XLSX)

S4 Table Expression and annotation of genes predicted to be directly controlled by the CebR repressor. (XLSX)

## Abbreviations

AFEX: Ammonia Fiber Expansion
CAZy: Carbohydrate-Active Enzyme
CBM: Carbohydrate-Binding Module
FP: Filter-Paper
GH: Glycoside Hydrolase
LPMO: Lytic Polysaccharide Monooxygenase
